# Accuracy and feasibility of a free-breathing cine technique sparsely sampled with iterative reconstruction for rapid evaluation of left ventricular function in adults and children

**DOI:** 10.1186/1532-429X-17-S1-P3

**Published:** 2015-02-03

**Authors:** Juliano L Fernandes, Luciana A Fioravante, Michael O Zenge, Michaela Schmidt, Mariappan S Nadar, Ralph Strecker

**Affiliations:** 1 Cardiovascular Imaging, Jose Michel Kalaf Research Institute, Campinas, Brazil; 2 Radiologia Clinica de Campinas, Campinas, Brazil; 3 Siemens Corporate Research, Princeton, NJ, USA; 4 Siemens Ltda, Sao Paulo, Brazil; 5 Siemens Healthcare, Erlangen, Germany

## Background

Despite being the gold standard for left ventricular function evaluation, traditional SSFP sequences still require multiple breath-holds and a relatively long scan time. This extends total duration of the exam, especially in children or adults with impaired cardiopulmonary capacity. We sought to compare a technique employing a prototype sparse sampling with iterative reconstruction (SSIR) for rapid evaluation of the left ventricle (Liu J et al. ISMRM; 2012) in a single breath-hold and free-breathing (FB) compared to traditional SSFP sequences and temporal parallel imaging methods.

## Methods

Patients undergoing routine clinical CMR for iron deposition evaluation with normal myocardial iron were evaluated with five different short axis cine techniques: traditional SSFP with 4-5 breath-holds, temporal parallel acquisition technique with 6-factor acceleration (TPAT6) in two breath-holds, SSIR with acceleration factor of 11.5x in one breath-hold, TPAT6 FB and SSIR FB. Left ventricular global function, diastolic and systolic volumes and mass were compared using repeated measures ANOVA and Bland-Altman plots. Traditional SNR and CNR were not used due to inherent properties of SSIR methods but a comparison of the ratio between left ventricular cavity blood and myocardium signal was performed to assess contrast among borders used for ROI tracings.

## Results

**Figure 1:**
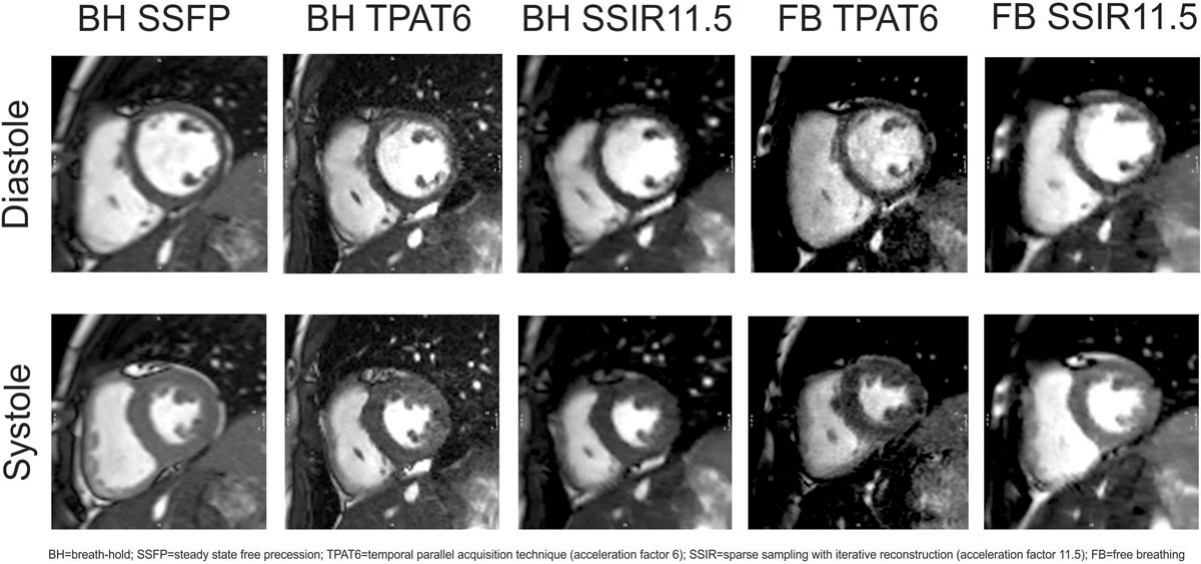


Fifty consecutive patients (29 adults, 21 children, mean age 26.6±14.2, 42% males) completed all five sequences. An example of images at diastole and systole is demonstrated in Figure 1. Using the SSFP sequence as reference, no significant differences were seen among the five sequences for ejection fraction (Table 1). The SSIR FB sequence resulted in similar systolic volume compared to SSFP but underestimated diastolic volume by 5.5% and overestimated total mass by 6.7%. Compared to the breath-hold SSIR sequence, the SSIR FB technique showed no significant differences regarding any of the parameters measured except for inferior ventricular mass. In the comparison of blood/myocardium signal intensity ratio, both TPAT6 and SSIR BH sequences demonstrated better contrast compared to SSFP, while the FB SSIR technique resulted in similar ratios. For children under the age of 18, the SSIR FB showed only significant differences compared to SSFP in terms of ventricular mass with similar values for global function and mass.

**Table 1:** Values represented as means (95% confidence intervals); signal ratio in % compared to SSFP

Sequence		Ejection Fraction (%)	Diastolic Volume (mL)	Systolic Volume (mL)	Mass (g)	Blood/myocardium SI ratio
BH SSFP	Mean	67.3 (65.3 to 69.3)	126.2 (116 to 136)	40.7 (37 to 45)	86.9 (80 to 94)	3.9 (3.7 to 4.3)

BH TPAT6	Mean	68.3 (66.3 to 69.9)	116.8 (108 to 126)	37.5 (33 to 42)	93.9 (86 to 102)	4.9 (4.5 to 5.3)
	
	Mean Differences	-1.0 (-3.6 to 1.6)	9.4 (3.9 to 14.9)	3.1 (-0.5 to 6.8)	-7.1 (-12 to -2.2)	23% (7 to 38)
	
	P	1.0	0.0001	0.14	0.001	0.001

BH SSIR11.5	Mean	66.7 (64.9 to 8.5)	114.6 (106 to 123)	38.8 (35 to 443)	97.0 (90 to 104)	4.8 (4.3 to 5.3)
	
	Mean Differences	0.56 (-2.1 to 3.2)	11.6 (5.7 to 17.5)	1.8 (-1.8 to 5.4)	-10.2 (-15.6 to -4.8)	22% (6 to 38)
	
	P	1.0	<0.0001	1.0	<0.0001	0.002

FB TPAT6	Mean	67.2 (65.6 to 68.9)	122.2 (113 to 131)	38.8 (35 to 443)	97.0 (90 to 104)	4.8 (4.3 to 5.3)
	
	Mean Differences	0.56 (-2.1 to 3.2)	11.6 (5.7 to 17.5)	1.8 (-1.8 to 5.4)	-10.2 (-15.6 to -4.8)	22% (6 to 38)
	
	P	1.0	0.18	1.0	1.0	0.0002

FB SSIR11.5	Mean	65.2 (63.5 to 66.9)	119.2 (110 to 128)	42.1 (37 to 47)	92.6 (86 to 100)	3.9 (3.1 to 4.7)
	
	Mean Differences	2.0 (-0.5 to 4.5)	7.0 (2.9 to 11.1)	-1.4 (-4.7 to 1.9)	-5.8 (-9.9 to -1.6)	-2% (-6 to 28)
	
	P	0.22	0.0001	1.0	0.002	1.0

BH=breath-hold; SSFP=steady state free precession; TPAT6=temporal parallel acquisition technique (acceleration factor 6); SSIR=sparse sampling with iterative reconstruction (acceleration factor 11.5); FB=free breathing

## Conclusions

The use of a rapid prototype SSIR cine sequence in FB provides feasible and accurate evaluation of left ventricular function and volumes, with small deviations in diastolic volume and mass compared to traditional SSFP sequences. The same results were also observed in the pediatric group, demonstrating the possible usefulness of the sequence for fast evaluation of both adults and children in routine practice.

## Funding

N/A.

